# Addressing Racism in Medical Education: A Longitudinal Antiracism Discussion Curriculum for Medical Students

**DOI:** 10.1007/s40670-023-01788-x

**Published:** 2023-04-28

**Authors:** Daniel Carrera, Christian Tejeda, Preeti Kakani, Jason Napolitano

**Affiliations:** David Geffen School of Medicine, University of California, Los Angeles, Los Angeles, CA USA

**Keywords:** Medical education, Antiracism, Common book, Discussion curriculum

## Abstract

Although recent efforts have been engaged to combat bias in medical education, minimal attention has been dedicated to developing antiracism curricula for medical students. We developed a year-long discussion curriculum for 175 first-year medical students centered around Ibram X. Kendi’s *How to be an Antiracist*. The discussion curriculum consisted of six, 2 hour seminars. We evaluated students’ perceptions regarding discussing and actively addressing racism. Students reported an improved ability and comfort to discuss and address racism within healthcare settings. These data suggest that antiracism discussion curricula may be effective for training medical students to address racism in their future careers.

## Background

Structural racism, or the totality of ways in which societies foster racial discrimination through mutually reinforcing inequitable systems, observable in education, employment, housing, criminal justice, and health care, contributes to significantly higher morbidity and mortality among racial minorities [1–3]. The impact of racism on patients is well chronicled, and it is increasingly recognized that racial bias adversely influences medical decision-making, reduces access to critical medical treatment, and worsens health disparities [1–7]. Healthcare providers, along with medical trainees, have a responsibility to address the effects of racism on patients [4].

Although recent efforts have begun to engage providers in identifying biases and strategies to achieve allyship, minimal attention has been dedicated to developing discussion-based antiracism curricula for medical students. Additionally, there is limited research surrounding comfort levels among medical students in discussing and confronting racism in the clinical setting [5]. The police and civilian killings of unarmed African Americans including Daniel Prude, Breonna Taylor, George Floyd, Ahmaud Arbery, and multiple others in 2020 and subsequent national racial justice protests further laid bare the systemic racism present in the USA and galvanized the development of antiracism policy and training across the healthcare system [8, 9]. In response to the call for institutional allyship and change, student leaders and faculty at the David Geffen School of Medicine at UCLA identified the need for antiracism training for first-year medical students and developed a discussion curriculum centered around the themes of a “common book,” *How to Be an Antiracist* by Ibram X. Kendi.

## Methods

All first-year medical students (M1s; *n* = 175, Table 1) participated in six, 2-h “common book” discussion seminars as a stand-alone curriculum throughout the academic year. Each seminar consisted of three sections: a large-group lecture delivering key educational content on assigned chapters (20 min), an extended small-group discussion session where M1s discussed the themes of assigned chapters and relevant cases centered around racism in health care (80 min), and a large-group debrief (20 min). Seminars occurred once per block in the first-year curriculum, corresponding to approximately once every 1–2 months, and groups remained consistent. Seminars were held online via Zoom. Oral consent for the study was obtained during the first seminar.

**Table 1:** Participant demographics

**Demographics (*n* = 175)**	**Number of respondents (% of total)**
Gender	
Female	109 (62.3%)
Male	64 (36.6%)
Non-binary	2 (1.1%)
Age	
18–21	7 (4%)
22–25	124 (70.9%)
26–29	33 (18.9%)
30+	11 (6.3%)
Racial or ethnic identity	
Black or African American	23 (13.1%)
White	58 (33.1%)
Asian	58 (33.1%)
Hispanic, Latinx, or Spanish Origin	25 (14.3%)
Other	11 (6.3%)

The first large group discussion was led by faculty and student leaders. The faculty began with an overview of nonviolent communication and tools for difficult conversations [10]. Then, the student leaders reviewed key terms associated with the assigned chapters. M1s were asked brief questions related to these terms and responses were recorded anonymously via Mentimeter, an interactive online polling software, to be displayed on the screen. Lastly, the large group discussion ended by reviewing Brave Space Rules, adapted by AWARE-LA’s “Communication Guidelines for a Brave Space” [11], aimed at promoting healthy discussion.

The small group discussion was led by 1–2 student facilitators with a group of 8–10 M1s. Student facilitators consisting of M2s–M4s were selected via an application process and were required to attend an Antiracism Facilitator workshop led by the UCLA Center for Education, Innovation and Learning in Sciences. The workshop discussed topics such as privilege, racial bias, and facilitation skills with a roleplay exercise where student facilitators were observed leading a small group discussion. Before each seminar, facilitators were provided a facilitator guide created by the student leaders that was inspired by a book club kit from the author [12] and consisted of 2–3 discussion questions per chapter with supplemental facilitator notes that offered key educational concepts. M1s received the discussion questions before each seminar without the notes. Facilitators were not required to cover every question but were encouraged to discuss at least one question per assigned chapter. Facilitators were instructed to allow for flexibility and encourage M1s to share their perspectives within a brave space. Faculty would intermittently join groups to monitor progress of the discussions.

The final large group debrief was again led by the student leaders. M1s were asked to provide takeaway points that could be provided anonymously via Mentimeter. The student leaders researched and compiled external resources to create an “Antiracism Toolkit,” which was offered to the M1s and consisted of videos and supplementary links. Lastly, every seminar ended with a breathing meditation exercise and a list of student support campus resources (Table 2).

**Table 2:** Two-hour discussion seminar structure

**1. Large group lecture & discussion (20 min)**	**2. Small group discussion (80 min)**	**3. Large group reflection (20 min)**
• Tips on nonviolent communication and response • Define key terms from assigned chapters • Use Mentimeter to elicit anonymous responses • Cover Brave Space Rules	• “Common book” student facilitators lead discussion with groups of 8–10 M1s • Discuss prepared questions which focus on key themes and connection of Kendi’s ideas with medicine	• Deliver key takeaway points from the discussion • Provide antiracism toolkit for further learning • Provide external resources for support • Breathing meditation exercise

M1s completed an anonymous 20-question survey generated in-house via Google Forms before the first seminar and completed the same survey after the sixth seminar. The survey utilized a 6-point Likert scale to gather quantitative data about students’ perceptions of their own understanding of key concepts, comfort levels, personal motivations, and ability to identify strategies regarding discussing and actively addressing racism and attitudes about the importance of addressing racism in medical education. 175 students completed the initial survey, and 110 students completed the second survey. Group responses were analyzed using an unpaired *t* test to assess change over time.

## Results

Questionnaire items exhibiting a statistically significant difference between the pre- and post-survey were those related to identifying strategies to discuss and address racism within health care, as well as comfort with discussing and addressing racism in a healthcare setting. For example, M1s reported greater comfort discussing racism with patients (difference = 0.61; *p* < 0.001), greater ability to identify strategies for discussing racism with patients (difference = 0.78; *p* < 0.001), and greater comfort addressing racism in patient care (difference = 0.23; *p* = 0.042). In addition, there was a statistically significant increase in student-reported understanding of the concept of race (difference = 0.20; *p* = 0.024).

Notably, there were no significant differences between the pre- and post-survey results in M1s’ understanding of racism and antiracism, as well as personal motivation to discuss and address racism. However, these survey items were rated higher at baseline compared to survey items that exhibited a significant difference between surveys. Survey results are summarized in Table 3.

**Table 3:** Summary of pre- and post-survey results

**Survey item**	**Pre-survey mean (*n* = 175)**	**Post-survey mean (*n* = 110)**	**Difference**	***p* value**
I understand the concept of race	5.15	5.35	0.20	0.024*
I understand how race can impact an individual’s health and the medical care that they receive	5.41	5.51	0.10	0.211
I understand the concept of racism	5.39	5.50	0.11	0.140
I understand how racism can impact an individual’s health and the medical care that they receive	5.51	5.53	0.02	0.849
I understand the concept of antiracism	5.18	5.32	0.14	0.092
I understand how antiracism can impact an individual’s health and the medical care that they receive	5.24	5.37	0.13	0.103
I am able to identify strategies for discussing racism with patients	3.94	4.72	0.78	< 0.001***
I am able to identify strategies for discussing racism with other healthcare professionals	4.23	4.75	0.52	< 0.001***
I am able to identify strategies for actively addressing racism in patient care	4.31	4.73	0.42	< 0.001***
I am able to identify strategies for actively addressing racism in interactions with other healthcare professionals	4.31	4.72	0.41	0.001**
I feel comfortable discussing racism with patients	3.92	4.53	0.61	< 0.001***
I feel comfortable discussing racism with other healthcare professionals	4.42	4.77	0.35	0.002**
I feel comfortable actively addressing racism in patient care	4.54	5.77	0.23	0.042*
I feel comfortable actively addressing racism in interactions with other healthcare professionals	4.43	4.75	0.32	0.007**
I feel personally motivated to discuss racism with patients	4.82	4.98	0.16	0.213
I feel personally motivated to discuss racism with other healthcare professionals	5.15	5.06	− 0.09	0.424
I feel personally motivated to actively address racism in patient care	5.45	5.29	− 0.16	0.079
I feel personally motivated to actively address racism in interactions with other healthcare professionals	5.34	5.18	− 0.16	0.102
It is important that medical schools integrate formal discussions of race, racism, and antiracism into their curricula	5.80	5.70	− 0.10	0.191
In order for future physicians to deliver adequate health care, it is important that as medical students, they understand and formally discuss race, racism, and antiracism as part of their curriculum	5.79	5.70	− 0.09	0.240

^*^p < 0.05; **p < 0.01; ***p < 0.001

## Discussion

Our common book curriculum addressed various components of racism and antiracism within the healthcare setting. The curriculum had two key effects: helping M1s identify strategies to address these issues and improving comfort in doing so. To help students build strategies, we provided tips on nonviolent communication, Brave Space guidelines, and an antiracism toolkit. Providing structured communication tools allows students to discuss these issues in a meaningful way and together brainstorm strategies to address racism in a clinical context. Our curriculum also improved the comfort levels of students when discussing these issues. Creating a brave space allowed students to share their thoughts and opinions openly. Further, having near-peer facilitators for the small group discussions minimized power dynamics between group leaders and members. By providing regular seminars throughout the year, we normalized these discussions to become an integrated part of M1s’ medical training. Repeated exposure to these discussions may ease discomfort, allowing the M1s to be more likely to discuss these topics with patients and other healthcare professionals.

From the survey results, M1s rated the importance of addressing racism during medical school and in the healthcare setting highly, suggesting that they value having these discussions before entering clinical training. Although reported comfort levels and ability to identify strategies improved between the pre- and post-survey, the domains of understanding and motivation did not show a significant change. Interestingly, the pre-survey questions regarding understanding and motivation were marked highly. This suggests that the M1s felt they already had a strong understanding and motivation to address racism and antiracism prior to the discussion curriculum. It should be noted that M1s had read the book before this initial survey. While this study design was implemented to measure the effect of the discussion curriculum rather than that of reading the book, a preliminary survey administered prior to reading the book may have added additional insight to the results but was forgone due to logistical challenges of administering a survey to M1s prior to matriculation.

In conclusion, our curriculum design of large and small group discussion seminars centered around a “common book” can help first-year medical students build proficiency in addressing racism and antiracism in health care. This study had several limitations. First, not all students completed the post-curriculum survey which may have diminished the potential effect of curriculum participation. This may have been addressed by administering the post-curriculum survey before rather than after the breathing meditation exercise while students are still fully engaged. Second, individual identifiers were not collected so individual trends could not be assessed. While we assessed group trends, it is possible that certain subgroups may have had a differential effect, for example, based on gender and race. In the future, this study could be expanded to provide students with practice navigating issues of racism through standardized patient encounters. The curriculum could also be adapted for M2s–M4s, though careful planning and consideration would be required due to the variability of each class year’s unique schedule. Additionally, future studies are needed to assess the long-term impact of antiracism curricula on the quality of care, patient-physician relationships, and health disparities. Ultimately, this pilot study was a first step toward building a robust and sustainable antiracism curriculum for medical students.

## Data Availability

Any data or materials used in this study can be de-identified as needed and made available on request.
